# 700 Use of Antibiograms and Changes in Bacterial Resistance Patterns

**DOI:** 10.1093/jbcr/irac012.254

**Published:** 2022-03-23

**Authors:** Josephine D'Abbondanza, Natalia Ziolkowski, Sarah Rehou, Shahriar Shahrokhi

**Affiliations:** University of Toronto, Toronto, Ontario; University of Toronto, Toronto, Ontario; Sunnybrook Health Sciences Centre, Toronto, Ontario; Sunnybrook Health Sciences Centre, Toronto, Ontario

## Abstract

**Introduction:**

Infection is a leading cause of death in burn patients. With an increase in resistance patterns, management of these infections has become progressively difficult. Antibiograms, a summary of susceptibilities to bacteria in a given institution or area, are often used to guide empiric treatment of infections. However, inappropriate prescribing and use of empiric antimicrobials may greatly impact the incidence of resistance. Currently, we do not know the patterns of antibiotic use since the introduction of institutional antibiograms or associated changes in antibiotic resistance. The objective of this study is to describe trends in antibiotic susceptibilities in burn patients in Canada pre- (PrA) and post-introduction (PoA) of antibiograms.

**Methods:**

We performed a retrospective review of patients admitted to an ABA-verified Burn Centre 2 years pre- (2013-2014) and post-introduction (2016-2017) of institutional antibiograms and started on broad-spectrum antibiotics (meropenem, piperacillin-tazobactam, and/or vancomycin).

**Results:**

A total of 864 patients were admitted during the study period (*n*=420 PrA and *n=*444 PoA). Average age, % total body surface area (%TBSA), and length of stay were similar between cohorts. Administration of empiric meropenem increased (43.2% vs. 56.8%) and piperacillin-tazobactam decreased (60.6% vs. 39.4%), which was significant (*p*=0.002). The use of vancomycin was unchanged. There was a significant decrease in the overall use of empiric antibiotics (*p*=0.002) since the inception of antibiograms, with a significant improvement in culture and sensitivity (C&S) testing within 5 days of starting empiric antibiotics (*p*=0.002). There was no significant difference in use of targeted antibiotics pre- or post-antibiogram introduction.

**Conclusions:**

Our study demonstrates that since the inception of antibiograms, there has been a significant decrease in overall use of empiric antibiotics and improvement in acquiring C&S within 5 days. However, these antibiotics were not always targeted to the appropriate organism and therefore may contribute to multi-drug resistant organisms in a burn population.

